# Multi-Scale Simulations Provide Supporting Evidence for the Hypothesis of Intramolecular Protein Translocation in GroEL/GroES Complexes

**DOI:** 10.1371/journal.pcbi.1000006

**Published:** 2008-02-29

**Authors:** Ivan Coluzza, Alfonso De Simone, Franca Fraternali, Daan Frenkel

**Affiliations:** 1 Department of Chemistry, University of Cambridge, Cambridge, United Kingdom; 2 Randall Division of Cell and Molecular Biophysics, King's College London, London, United Kingdom; 3 FOM Institute AMOLF, Amsterdam, The Netherlands; University of California San Diego, United States of America

## Abstract

The biological function of chaperone complexes is to assist the folding of non-native proteins. The widely studied GroEL chaperonin is a double-barreled complex that can trap non-native proteins in one of its two barrels. The ATP-driven binding of a GroES cap then results in a major structural change of the chamber where the substrate is trapped and initiates a refolding attempt. The two barrels operate anti-synchronously. The central region between the two barrels contains a high concentration of disordered protein chains, the role of which was thus far unclear. In this work we report a combination of atomistic and coarse-grained simulations that probe the structure and dynamics of the equatorial region of the GroEL/GroES chaperonin complex. Surprisingly, our simulations show that the equatorial region provides a translocation channel that will block the passage of folded proteins but allows the passage of secondary units with the diameter of an alpha-helix. We compute the free-energy barrier that has to be overcome during translocation and find that it can easily be crossed under the influence of thermal fluctuations. Hence, strongly non-native proteins can be squeezed like toothpaste from one barrel to the next where they will refold. Proteins that are already fairly close to the native state will not translocate but can refold in the chamber where they were trapped. Several experimental results are compatible with this scenario, and in the case of the experiments of Martin and Hartl, intra chaperonin translocation could explain why under physiological crowding conditions the chaperonin does not release the substrate protein.

## Author Summary

Chaperonin complexes capture proteins that have not yet reached their functional (“native”) state. Non-native proteins cannot perform their function correctly and threaten the survival of the cell. The chaperonins help these proteins to reach their native state. The prokaryotic GroEL-GroES chaperonin is an ellipsoidal protein complex that is approximately 16 nm long. It consists of two chambers that are joined at the bottom. Interestingly, protein repair by this chaperonin is not a one-step process. Typically, several capture and release steps are needed before the target protein reaches its native state. It is commonly assumed that substrate proteins cannot translocate, i.e., move inside the complex from one chamber to the other. In the absence of translocation, proteins that have not yet reached their functional conformation have to be released into the cytosol before being recaptured by a chaperonin. We present multi-scale simulations that show that it is, in fact, surprisingly easy for substrate proteins to translocate between the two chambers via an axial pore that is filled with disordered protein filaments. This finding suggests that non-native proteins can be squeezed like toothpaste from one chamber to the other: the incorrect structure of the protein is broken up during translocation and the protein has an increased probability to find its native state when it reaches the other chamber. The possibility for intra-chaperonin translocation obviates the need for a potentially dangerous release of non-native proteins.

## Introduction

**Figure 1:**
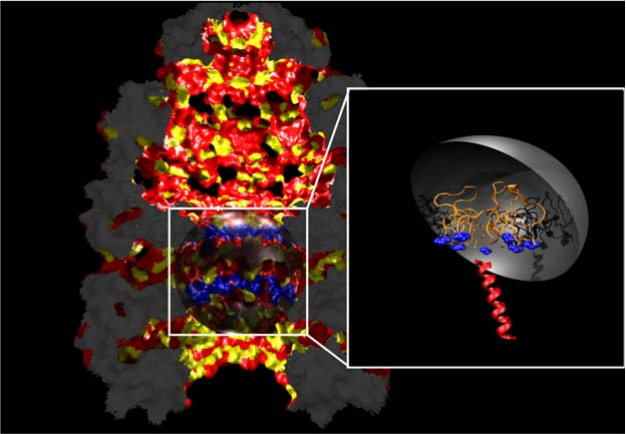
Space-filling representation of the X-Ray structure of the GroEL/GroES/ADP complex [5]. Colours represent the type of surface: all hydrophobic amino acids (A, V, L, I, M, F, P, Y) are in *yellow*, while the polar ones (S, T, H, C, N, Q, K, R, D, E) are *red*. The sphere in the equatorial region has a radius of 40 Å and models the cavity between the cis and the trans chamber. In the inset we show the actual simulation setup, consisting of: the confining sphere, the chains (orange) anchored the GroEL amino acids in the equatorial region (blue), and a test alpha helix (red) allowed only to rotate around the center of mass, and to translate.

**Figure 2:**
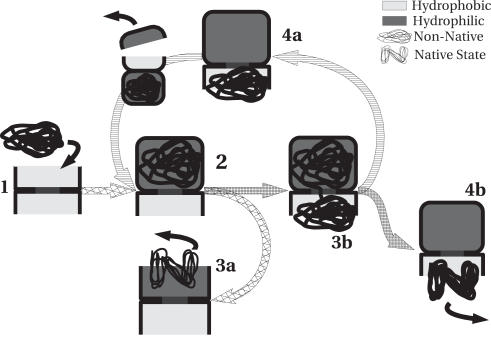
The present simulations suggest that non-native proteins may reach their native state either by the standard “intra-chamber” folding or by translocation through the equatorial region. The two pathways are shown in the schematic drawing above. In the initial configuration (1), the chaperonin barrel is open and exposes a hydrophobic rim for binding partially folded proteins. After a non-native protein is captured, the GroEL-GroES complex closes (i.e., the barrel gets capped) (2). After that, the protein can either refold in the original barrel (3A) or, if their structure is far from native [6], translocate to the other side (3B). The early stages of translocation cost free energy, as the protein must locally unfold to initiate the translocation. This implies that the translocation route will be preferentially followed by relatively unstable non-native conformations. The gain in free energy as a result of folding facilitates the subsequent translocation process, when the protein enters the other barrel of the chaperonin complex (4). If, after translocation, the protein is still in a non-native state, it will remain trapped, as the surface of the open barrel can bind to the hydrophobic surface of non-native proteins. In this way the folding cycle can start again, with the capping of the second cavity and the opening of the first. The process (shuttling) continues until folding is completed.

Proteins that have not yet folded to their native state may interfere with the machinery of the cell. For this reason, prokaryotic and eukaryotic cells have evolved special macro-molecular “chaperone” complexes that capture and refold partially folded proteins, thereby preventing them from indulging in cellular mischief [1],[2],[3,]. An important class of chaperone complexes are the cage chaperones or *chaperonins*. These complexes can efficiently trap partially folded proteins in a cavity that is barely larger than the target protein, and assist in the folding of an entire class of proteins with different amino acid sequences. Hence, the chaperonin is able to distinguish partly folded states from the native state, independently of the specific amino-acid sequence. It is important to stress that in the presence of molecular crowding (similar to the one present in a cell) the chaperonin complex has been demonstrated to not release the substrate protein before it reaches the native state [4]. Below, we report a detailed numerical study of protein dynamics inside the so-called GroEL-GroES chaperone complex. The GroEL complex consists of two barrel-shaped protein complexes joined at the bottom (see Figure 1). Non-native proteins can be captured in an open GroEL “barrel”. The GroES “lid” can then cap a protein-containing barrel, thereby initiating the refolding process. After about 15 seconds and several refolding cycles, the GroES cap is released and the other barrel is capped (if it contains a protein). A single “cycle” of the GroEL-GroES chaperone hydrolyses seven ATPs [5]. This energy is presumably used to compress the protein in a smaller, more hydrophilic GroEL cavity, thus increasing the thermodynamic driving force to expel this protein. Recently we reported simulations of the kinetics of chaperone-induced protein refolding, using a lattice model for the GroEL-GroES complex [6]. This study suggested that proteins may refold either inside the cavity in which it has been captured or, surprisingly, by translocating from one barrel of the GroEL dimer to the other (see Figure 2). This second route is unexpected because it is generally believed that proteins cannot cross the equatorial plane that separates the joined GroEL barrels [7],[8],[9].

In the present paper we use atomistic and mesoscopic simulations to test whether such a translocation scenario is compatible with the available structural information on the GroEL complex. Our simulation studies focus on the equatorial regime of the GroEL complex that might be expected to act as a barrier against translocation. Crystallographic studies indicate that most protein units in the chaperonin complex have a fairly rigid structure both in the open and closed configurations [5]. However, low-resolution small-angle neutron scattering experiments [7] and cryo-electron microscopy [8],[9] indicate the presence of disordered residues in a central cavity of the equatorial region. These chains do not show up in the X-ray crystallographic structure of the GroEL complex.

The presence of disordered protein chains in the pore that joins the two GroEL chambers will certainly affect the permeability of the equatorial plane, but they need not block translocation. There are, in fact, examples [10] where disordered protein chains near a pore act to enhance the selectivity of the translocation process. Interestingly, the chemical composition of the disordered chains in the GroEL complex is similar to that of chains in known translocation channels in the nuclear pore complex.

## Results

**Figure 3:**
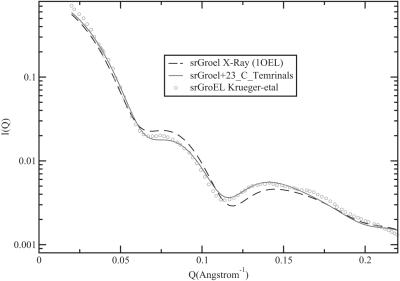
Validity test of the full atomistic run. The plot shows the experimental scattering intensity of solution of single ring GroEL obtained with SANS (o). Fitted over the data with the program Cryson [12], we plotted the scattering intensity of the MD equilibrated structure (___) and of the X-Ray structure obtained by Brag et al. [26] (- - -). The fit of the simulated data is significantly better than the one from the X-Ray structure indicating that the representation of the C-Terminal chains is accurate.

**Figure 4:**
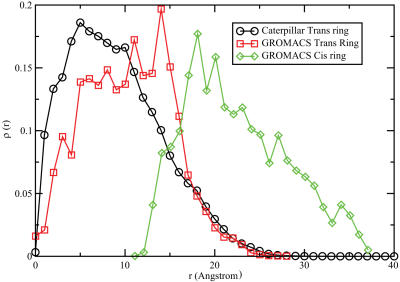
Plot of the density profile ρ[*r*] of the filament's *C*_α_ as a function of the radial distance from the central axis of the hole *r*. The data are obtained with the caterpillar model of the chains in the trans ring (circles), and with GROMACS full atomistic simulations for the trans (squares) and cis (diamonds) chains. The remarkable overlap between the distributions indicates that we do reproduce the static picture of a blocked hole. The density distribution generated by the coarse-grained model is more peaked than the one generated in the atomistic simulations. This indicates that, if anything, the coarse-grained model will overestimate the extent to which the disordered peptide chains hinder translocation. The coarse-grained model predicts that the chains in the cis region are far from the center of the hole and do not impede the passage of an α-helix.

We have performed fully atomistic and coarse-grained simulations that do reproduce the structural data of [7], and allowed us to bridge the computational cost of computing the translocation free energy barrier of a short alpha helix. For the fully atomistic simulations in explicit water we used the GROMACS Molecular Dynamics (MD) simulation package [11]. MD simulations of 10 ns were performed on the structure of the central region at which time the system had equilibrated (Figure S1). In order to compute the scattering profile we used the program CRYSON from Svergun et al. [12]. Figure 3 shows that the neutron-scattering form factor computed on the basis of equilibrated structure of the trans ring agrees well with the experimental data of Krueger et al. [13]. Interestingly, the simulations show that chains on the cis ring do not obstruct the passage between the two GroEL chambers (see Figure 4 and Figure S2). The chains in the trans ring fluctuate in a region between 5 and 15 Å from the center, in agreement with hollow-cylinder model proposed by Krueger et al. on the basis of their experimental data [13].

To compute the free-energy barrier for protein translocation, the MD approach described above would have been prohibitively expensive. We therefore performed Monte Carlo simulations on a suitably coarse-grained model for the GroEL complex. We focused on the structural fluctuations within a spherical region (diameter 40 Å) around the trans side of the equatorial cavity (Figure 1), because the cis chains did not appear to represent an obstacle to translocation. The disordered chains in the cavity (22 monomeric units long) were rigidly anchored on a circular rim around the trans hole of ∼30 Å radius (Figure 1 inset). To this end, we represented all peptide backbones using a model that keeps track of the positions of 5 distinct types of backbone atoms (H, N, *C*_α_, C, and O). Side chains are represented as hard spheres with a radius of 2.5 Å, centred on the *C*_α_ atoms. Neighbouring spheres along the chain are allowed to overlap (see Figure S3). We used this coarse-grained model to estimate the free-energy cost associated with the insertion of a short and rigid helix, 21 monomeric units long, in the region of disordered protein chains. We sampled the free energy as a function of a reaction coordinate *Q_s_* that measures the progress of the translocation process. *Q_s_* is defined as the total number of *C*_α_ atoms that have passed the entrance of the trans ring. We define the entrance as a plane through the average position of the hydrogen atoms in the anchoring amino acid of the chains. In order to translocate, a protein must first “find” the translocation hole. From our study of a lattice model GroEL [6], we know that this first step is relatively easy. The key question is therefore whether or not the free-energy cost for the subsequent translocation is prohibitive. The present calculations address this issue by computing the free energy difference involved in moving an α-helix from the entrance of the pore region to the inside. Of course, the free-energy barrier depends on the interaction between the α-helix and the disordered chains that consist mainly of *Gly* and *Met*.

### Analysis

We start by considering a very naive estimate that has the advantage that it is based on the fully atomistic simulations. From these simulations, we know the density profile of *C*_α_ atoms in the trans ring (see Figure 4). If, in the spirit of the Flory model, we assume that the density fluctuations of independent polymer Kuhn segments are Poisson distributed, we can estimate the probability *P*_0_ that a tube with the diameter of an α-helix contains no *C*_α_ atoms at all. This would lead us to an estimate of the free energy barrier that is equal to −*kT*ln*P*_0_. Using the density profile of Figure 4 and an estimate [14] for the persistence length of a protein filament, we obtain a translocation barrier of approximately 4 *k_B_T*. If we make the (unrealistic) assumption that all *C*_α_'s in a single chain are fully correlated, then we estimate the barrier height to be only 1 *k_B_T*, which should be a significant underestimate. To see whether such a rough estimate is at all reasonable, we can repeat the same procedure for the coarse-grained model where we can also perform direct free-energy calculations. To be consistent with the previous case, we assume that the there are only excluded-volume interactions between the (mainly *Gly*) chains and the helix residues. In terms of the interaction matrix of [15] this is equivalent to assuming that the helix consist entirely of *Thr* residues. Assuming all Kuhn segments fluctuate independently, we estimate the barrier to be 4 *k_B_T*, and the assumption of fully correlated fluctuations will again yield an estimate of order 1 *k_B_T*. The good agreement between the fully atomistic and coarse grained estimates is, of course, somewhat fortuitous, in view of the fact that the two density distributions are not identical. However, it suggests that the coarse-grained model may be of practical use.

Next, we compute the free energy barrier for the coarse-grained model system using the MC method described in the Methods.

**Figure 5:**
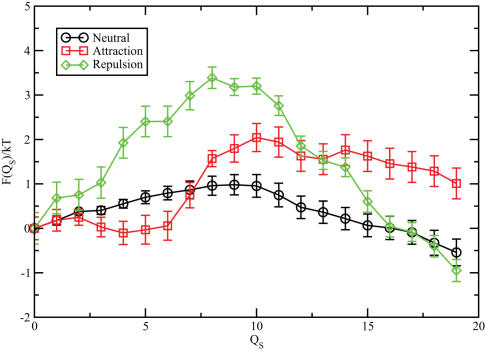
Translocation free energy as a function of the number of translocated amino acids *Q_s_*, for the different interaction scenarios. For steric interactions (circles), the profile is rather symmetrical and presents a small barrier of 2 *kT*. In the presence of mutual attraction between chains and the helix (squares), the barrier is very small and there is a symmetry breaking that favours the binding of the helix to the chains inside the cavity (small values of *Q_s_*). The final scenario is for repulsive helix-chains interactions (diamonds) where we have a symmetric barrier 4 *kT* high.

First we considered the case of pure steric interactions between both the chains and the helix. In Figure 5 we plot the free energy *F*(*Q*_S_) as a function of the reaction coordinate *Q_S_* that measures the number of *C*_α_'s that have entered the pore region. The plot shows a symmetric barrier with a height of approximately 2 *k_B_T*, which is surprisingly close to the estimate obtained assuming fully correlated fluctuations of protein segments. In other words: the chains tend to move as a whole in an out of the central area of the pore. This picture is supported by the snapshot of the pore region (Figure S4). The main conclusion that we can draw from the coarse-grained free-energy calculations is that the presence of seven protein chains in the central core region of the trans ring is not enough to obstruct translocation on steric grounds alone.

Of course, the interaction between a typical translocation protein segment and the ring chains is not purely steric. To consider the effect of both attractive and repulsive interactions, we consider the two cases separately. As the chains consist predominantly of *Gly*, we consider the scenarios that the interactions between the filament residues and the *C*_α_ atoms of the helix are all equal to the twice the average of all attractive (resp. repulsive) interaction energies of *Gly* in the Betancourt-Thirumalai interaction matrix [15] (−0.1*k_B_T* and +0.1*k_B_T*, respectively). The strength of attractive/repulsive interactions between the *C*_α_'s of the helix and the filament is therefore −0.2*k_B_T* (resp +0.2*k_B_T*). By taking an interaction that is double the average attractive/repulsive interaction strength, we are presumably modeling rather extreme cases that should put bounds on the actual translocation barrier.

Figure 5 shows the computed free-energy barriers for translocation in the case of attractive (resp. repulsive) interactions. The translocation barrier is appreciably lower when the chains attract the α helix (2 *k_B_T*) than in the opposite limit (4.5 *k_B_T*). However, the most striking observation is that the barrier is quite small in either case - a barrier of 4.5 *k_B_T* can easily be crossed due to the action of thermal fluctuations.

In fact, in the case of attractive interactions, there is virtually no barrier for translocation. This absence of a barrier may provide a rationale for the experimental observation that Krueger et al. observed in their SANS experiments [13] that a non-native protein (DPJ-9) was partially sucked into isolated trans rings. If proteins can indeed translocate through the GroEL equatorial plane then this may also be relevant for the mechanism by which the GroEL/GroES chaperonin can help the refolding of proteins that are too big to be encapsulated. In such cases, portions of the protein could be attracted to the inside of the pore and perform either a complete or a partial translocation (Figure S5). According to [6] either process can enhance the refolding efficiency.

The translocation of encapsulated non-native proteins is most likely in cases where the initial structure is far native. The reason is two fold: first of all, for such conformation there should be a low free-energy cost associated with partial unfolding—a necessary first step in translocation. Secondly, non-native chains that are trapped in a hydrophilic cage tend to be compressed. They can lower their free energy by translocating out of the cage. The simulations of [6] suggest that the driving force for such translocation can be as much as 0.5 *k_B_T* per amino-acid residue. Such a free-energy gradient is enough to completely remove a small free-energy barrier that might oppose translocation (Figure S6).

## Discussion

In conclusion, our simulation results are not compatible with the assumption that the disordered protein chains in the cis or trans rings provide an effective barrier against translocation. The present findings may help explain a puzzling experimental finding concerning refolding experiments in the presence of crowding agents [4]. The experiments of [4] demonstrated that, under physiological crowding conditions, the substrate protein does not escape from the chaperonin until it has reached its native state. This phenomenon is difficult to reconcile with the standard scenario where a protein (folded or not) is expelled from the cis-chamber as another non-native protein binds to the ATP-trans chamber. However, if it is not *another* protein that binds to the hydrophobic rim of the trans chamber, but the original protein that has translocated from the cis-chamber (see Figure 2), then it becomes clear why non-native proteins are unlikely to escape. We stress that the present findings do not rule out the possibility that non-native proteins fold into the native state without translocation [16]—translocation is simply an added route for protein folding. Such a route maybe very important for proteins that folds co-translationally, where confinement in a optimal size tunnel is crucial for efficiently reaching the native state [17].Our simulations suggest that it would be interesting to carry out refolding experiments on GroEL with mutated chains that would strongly stick to each other (or that could be cross-linked). Such mutation would impede the translocation and should thereby reduce the efficiency of the GroEL/GroES complex.

## Materials and Methods

### Atomistic Molecular Dynamics

The flexible nature of this region prevented accurate X-ray determination of the chains filling the interconnecting pore. To obtain a full-atomistic model, the program MODELLER [18] has been used to generate a starting configuration of the chains missing in the X-ray structure (PDB code: 1AON) of the GroEL/GroES complex loaded with ADP. The reconstructed fragments (sequence KNDAADLGAAGGMGGMGGMGGM) are added at the C-term extremity of each monomeric building block of the chambers. In order to avoid steric clashes between the chains, the procedure has taken into account of the quaternary assembly of the chains. After the generation of the chains structures, three steepest-descent minimisations were performed, using the program GROMACS [11] (energy minimisation tolerance: 0.1, 0.05 and 0.01 kJ/mol^−1^nm^−1^). Molecular Dynamics (MD) simulations were subsequently performed with the GROMACS [11] package by using GROMOS96 force field with an integration time step of 2 fs. Non-bonded interactions were accounted for by using the particle-mesh Ewald method (grid spacing 0.12 nm) [19] for the electrostatic contribution and cut-off distances of 1.4 nm for Van der Waals terms. Bonds were constrained by LINCS [20] algorithm. The system was simulated in the NPT ensemble by keeping constant the temperature (300 K) and pressure (1 atm); a weak coupling [21] to external heat and pressure baths was applied with relaxation times of 0.1 ps and 0.5 ps, respectively. As we intended to simulate a solution at a pH-value of 7 the protonation states of pH sensitive residues were assigned as follow: *Arg* and *Lys* were positively charged, *Asp* and *Glu* were negatively charged and *His* was neutral. The protein's net charge was neutralised by the addition of Cl^−^ and Na^+^ ions. It would have been prohibitively expensive to simulate the entire chaperonin plus surrounding water. However, this was not necessary, as our aim was to study the structure and dynamics of the strongly fluctuating the equatorial rings, rather than the relatively rigid remainder of the GroEL “chamber”. We therefore immobilised the chamber atoms that are not directly connected to the pore chains. Of course, the equatorial chains were free to move and relax in the pore. In order to further reduce the number of degrees of freedom treated, we only considered water molecules (SPCE [22]) *inside* the GroEL chamber. We achieved this by imposing a strong repulsive external potential outside the GroEL chamber. Ignoring the water outside the cage is not an unreasonable simplification, as we found that the disordered chains were completely solvated by water molecules and never moved outside the atoms of the internal surface of the chamber. We assumed periodic boundary conditions only along the symmetry axis of the GroEL complex (“z-axis”).

### Coarse-Grained Monte Carlo Simulations

The Caterpillar model is a modification of the tube model of Maritan and co-workers [14],[23],[24]. The main differences are that we treat the structure of the backbone in more detail and that our scheme to account for self avoidance by means of bulky side groups is computationally cheaper than the approach of Maritan et al. who introduced a three-body interaction to achieve the same. The interaction between amino acids with different side chain *E_CA_* is given by the following expression
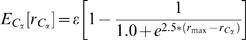
(1)where 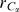 is the distance between nonadjacent *C*_α_ atoms in the protein and *r_max_* is the distance at which the potential has reaches half ε . For ε we use the 20×20 matrix derived with the method of Betancourt and Thirumalai [15].Although these interaction energies are strictly speaking neither energies nor free energies, they do provide a reasonable representation of the heterogeneity in the interactions between different amino acids. We modeled the hydrogen bonds between the hydrogen and the oxygen of the backbone with a 10-12 Lennard-Jones potential:
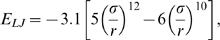
(2)where the minimum is at σ = 2.0 Å and *E_LJ_* = 3.1 *k_B_T*. The directionality of the hydrogen bond was taken into account by multiplying the Lennard-Jones potential by a pre-factor
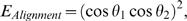
(3)where θ_1_ and θ_2_ are the angles between the atoms COH and OHN respectively. The large hard spheres centered on the *C*_α_ atoms ensure that the orientation factor is maximum only for angles close to π. Apart from rotations around the dihedral angles φ_1_ and φ_2_ (Figure S3), the backbone is rigid. We have verified that this model can indeed reproduce typical protein motifs such as alpha helices and beta sheets, depending on the amino-acid sequence.

### Folding

To sample the conformations of the protein chains anchored on the trans ring, we use two basic Monte-Carlo moves: branch rotation and an improved version of the biased Gaussian step [25], while for the translocating alpha helix we allow only translation moves and rotation around the center of mass.

## Supporting Information

Figure S1 Root mean square displacement of the C_α_ atoms of the equatorial chains compared to the initial condition. The time scale starts from 7 ns and goes all the way to 11 ns. The plateau demonstrates that the dynamics reached equilibrium.
(0.15 MB EPS)

Figure S2 Schematic representation of the model used for the GROMACS full atomistic simulations. The part of the protein that was kept constrained in space is shown in grey. The chains that were free to fluctuate are shown in light blue. The water molecules that fully solvated the protein complex are not shown. The axes are drawn to indicate the coordinate system used in the calculation of the filament density profiles.
(0.71 MB EPS)

Figure S3 Real-space representation of the backbone of the caterpillar model. The large blue sphere represents the self-avoidance area of the C_α_ atoms in with a radius of 2.5 Å. The H and O atoms interact through a 10-12 Lennard-Jones potential tuned with a quadratic orientation term that selects for alignment of the C H O and N atoms involved in a bond. The backbone fluctuates only around the torsional angles *Φ*_1_ and *Φ*_2_.
(1.11 MB EPS)

Figure S4 Real space snapshot of one configuration of the chains in the equatorial region equilibrated with the Monte Carlo simulation of the caterpillar model. The top and side view shows a fully blocked pore as seen in X-ray crystallography or Cryo-EM reconstruction.
(2.23 MB EPS)

Figure S5 Plot of translocation free energy *F* (*Q,Q_s_*) as a function of the number of Helix-chains contacts *Q* and of the number of translocated residues *Q_s_*. We plot *F* (*Q,Q_s_*) for an attractive (−0.2 *kT*) interaction between the alpha helix and the chains. In this scenario the alpha helix is pulled towards the middle of the hole, and it is subject to two choices, either to stay there surrounded by the chains (low values of *Q_S_* and high values of *Q*) or directly translocate (low values of *Q_S_*). The small barrier (<2 *k_B_T*) separating these two states suggests that the translocation can occur in one step (all the way down) or in two steps (first trapped for a while in the hole and then escaping).
(0.21 MB EPS)

Figure S6 Plot of translocation free energy *F′* (*Q_s_*) = *F* (*Q_s_*)−0.5*Q_S_* where *Q_s_* is the number of translocated residues, and *F* (*Q_s_*) is the translocation free energy with repulsive (0.2 *kT*) helix-chains interactions. The correction added to the free energy comes from the fact that the protein feels a gradient towards a a folded and translocated state. We extrapolated the coefficient −0.5 *k_B_T* per amino acids translocated, from previous work on lattice proteins [6].
(0.07 MB EPS)
